# VISTA Expression on Immune Cells Correlates With Favorable Prognosis in Patients With Triple-Negative Breast Cancer

**DOI:** 10.3389/fonc.2020.583966

**Published:** 2021-01-11

**Authors:** Xi Cao, Xinyu Ren, Yidong Zhou, Feng Mao, Yan Lin, Huanwen Wu, Qiang Sun

**Affiliations:** ^1^Department of Breast Surgery, Peking Union Medical College Hospital, Peking Union Medical College, Chinese Academy of MedicalSciences, Beijing, China; ^2^Department of Pathology, Peking Union Medical College Hospital, Peking Union Medical College, Chinese Academy of Medical Sciences, Beijing, China

**Keywords:** V-domain Ig suppressor of T-cell activation, triple-negative breast cancer, prognosis, tumor microenvironment, immune checkpoint

## Abstract

V-domain Ig suppressor of T-cell activation (VISTA), a newly discovered negative immune checkpoint, is thought to be related to immunotherapy resistance and may become a new immune therapeutic target. Here, we evaluated the expression of VISTA in a cohort containing 254 patients with untreated triple-negative breast cancer. The relevance of VISTA expression, clinicopathologic parameters, expression of other immune markers, and prognosis were investigated in the whole cohort. Genomic analysis of 139 triple-negative breast cancer (TNBC) patients from the cancer genome atlas (TCGA) was also performed. VISTA was expressed in the immune cells (ICs) and in the tumor cells (TCs) in 87.8% (223/254) and 18.5% (47/254) of the cohort, respectively. VISTA-positive ICs were associated with no lymph node metastasis (p < 0.001), American Joint Committee on Cancer (AJCC) stage I and II (p = 0.001) and basal-like subtype (p < 0.001). VISTA expression in ICs positively correlated with some tumor-infiltrating lymphocytes (TILs) types, particularly with the CD4^+^TILs, which was consistent with mRNA level analysis from the TCGA database. Survival analysis showed that patients with VISTA-positive ICs had prolonged relapse-free and overall survival compared with the negative ones, especially among T1-2N0 stage patients. Multivariate analysis showed that it independently predicted the prognosis. These data confirmed the regulatory role of VISTA in anti-tumor immunity, changed our perception of VISTA as a negative immune checkpoint, and suggested VISTA as a potential therapeutic target for TNBC.

## Introduction

Breast cancer (BC) is a major cause of death in women worldwide, with approximately 2.09 million new diagnoses and 626,679 BC-related deaths in 2018 (1). The incidence of BC among Chinese female from urban and rural areas has been increasing over the past 30 years (2, 3). According to the Chinese epidemiological investigation, 367,900 new BC cases and 97,972 BC-related deaths occurred in 2018 (4). Westernized lifestyles and changes in fertility patterns exacerbate BC development (5, 6), and the lack of early diagnostic tools and effective adjuvant therapies contribute to the higher mortality.

Triple-negative breast cancer (TNBC), defined as not expressing the estrogen receptor (ER), the progesterone receptor (PR), and human epidermal growth factor receptor 2 (HER2), accounts for nearly one fifth of all BCs (7). It is often diagnosed at a young age, has more aggressive biological features and lacks effective targeting treatment, resulting in a poor prognosis (8, 9). Nearly 20% of TNBC patients carry BRCA gene mutations, especially BRCA1 (10). Cytotoxic chemotherapy is the main systemic treatment. However, patients with metastatic TNBC respond poorly to rescue chemotherapy and have shorter overall survival than other BC subtypes (11). Adverse effects, including pain, nausea, vomiting, and even cardiovascular dysfunction severely affect the treatment of patients (12). Compared with the luminal subtype, TNBC is more immunogenic because it has more tumor-infiltrating lymphocytes (TILs) and higher mutational burden (13).

Recently, numerous clinical trials were performed to assess the effectiveness of immune checkpoint inhibitors. Blocking immune checkpoints improved the treatment response of various cancers. This may become an effective long-term treatment strategy for previously intractable cancers. Last year, the FDA approved atezolizumab (anti-PD-L1 inhibitor) combined with protein-bound paclitaxel for treating locally progressive or metastatic TNBC (14). However, inhibiting T-lymophocyte-associated antigen 4 (CTLA-4), programmed cell death-1 (PD-1) or PD-1-ligand 1 (PD-L1) resulted in lower than 30% response rates (15). Thus, new targets or combination therapy is needed.

V-domain Ig suppressor of T cell activation (VISTA) is a negative checkpoint regulator and type I transmembrane protein that shares an extracellular domain homologous with PD-L1 (16, 17).VISTA is expressed in myeloid cells, inflammatory monocytes, dendritic cells(DC), and lymphocytes including CD4 and CD8 T cells (18, 19). In multiple mouse models, the high expression of VISTA in myeloid cells plays a significant role in anti-tumor immunity (20, 21). A series of *in vitro* tests suggested that VISTA inhibited CD4^+^T cell activation by activating as both a co-inhibitory ligand and a receptor (17, 22). It also regulated naïve T cell quiescence and peripheral tolerance (23). VISTA has also been shown to non-redundantly inhibit T cell activation from the PD-1/PD-L1 pathway in murine models (24). Recent studies have found increased VISTA level in prostate cancer and metastatic melanoma after anti-CTLA4 (25) and anti-PD-1 treatment (26). This implied the potential for acquired immunotherapy resistance. These also showed that VISTA may become a new target, or it may be used in combination with other immunotherapy for cancer treatments.

Studies on VISTA in BC have been limited, and the correlation between VISTA and TNBC has not been reported. This study aimed to analyze the expression of VISTA in the TNBC tumor microenvironment (TME) and its relevance in relation to clinicopathological characteristics, other immune markers and clinical outcomes.

## Materials and Methods

### Patients and Tissue Microarray

Formalin-fixed paraffin-embedded (FFPE) tumor specimens were collected from 254 stage I-III TNBC patients and fabricated into a tumor microarray (TMA). To construct the TMA, we used the core needle to select 1 mm diameter areas containing tumor epithelial cells and tumor stroma from each FFPE section after hematoxylin and eosin (HE)-staining. Patients with *de novo* stage IV or inflammatory TNBC as well as those who received neoadjuvant chemotherapy were excluded. Patients who had incomplete medical records or adequate tumor and stromal contents for TMA cores were excluded.

All selected patients underwent curative surgery at our hospital from January 2011 to December 2014, and they subsequently received standard adjuvant therapy. After that, they were followed up regularly (median: 68 months; range: 3–103 months).

This study conformed to the Reporting Recommendations for Tumor Marker Prognostic Studies (REMARK criteria) (27) and the principles of the Declaration of Helsinki, approved by the committee on Human Research of Peking Union Medical College Hospital. The ethics committee on Human Research of Peking Union Medical College Hospital approved this study. Consent was obtained from all patients participating in the study.

### Immunohistochemistry

The sections of TMA were stained with VISTA, CD3, CD4, CD8, and CD19 antibodies. The rabbit IgG monoclonal antibody (D1L2G; dilution1:200, Cell Signaling Technology, USA) was used to detect the expression of VISTA. Human placental and tonsil tissues obtained from our hospital were used as positive control samples. SignalStain^®^Antibody Diluent #8112 and SignalStain^®^Boost (HRP, Rabbit) #8114 were used as diluent and detection reagents, respectively. The details of primary antibodies and immunohistochemistry were described in **Supplementary Table 1**. All sections were stained using the Leica stainer (Leica Biosystems, Germany) complying with the instructions. Two pathologists (XYR and HWW) independently reviewed the immunohistochemical staining and scored for each sample. The consensus of the two observers was more than 90%. Less than 10% of the sections had inconsistent results which were resolved *via* the joint evaluation of the particular tumor area.

### Assess of Immunostaining

The expression of VISTA was observed in the TME, including tumor cells (TCs) and immune cells (ICs). Tumor-infiltrated ICs were defined as the dendritic cells, myeloid cells, macrophages, and lymphocytes occupied the tumor. VISTA-positive TCs or ICs were those with membrane and/or cytoplasmic staining at any intensity. We dichotomized the VISTA expression in TCs and ICs into “negative” and “positive” groups, according to the median of raw proportion values. The median value of VISTA expression in ICs was 5%, which was consistent with the cutoff values recommended in previous studies (28, 29), while expression in tumor cells was 0%.

The percentages of CD3^+^TILs, CD4^+^TILs, CD8^+^TILs, and CD19^+^ TILs among the nucleated cells were also investigated as continuous values, and were dichotomized into “low” and “high” groups based on a median of 20, 10, 10, and 1%, respectively. TILs were evaluated according to recommendation of the International TILs Working Group 2014 (30) and were divided into “low” and “high” groups by median value of 5%.

The staining methods of BC related routine immune indexes were described previously (31, 32) and have been used during clinical practice in our hospital. Basal-like was defined as that ER, PR, and HER2 negative cancers that were positive for either EGFR or CK5/6.

### Analysis of Public Datasets

#### mRNA Expression from TCGA Database

Date on the gene-level RNA-seq expression date and clinicopathological information of patients were collected from the Breast Invasive Carcinoma (TCGA, PanCancer Atlas) (http://www.cbioportal.org/) database in January 2020. A total of 139 female patients with ER- and PR-and HER2- referred as TNBC were enrolled. Inclusion criteria included: (1) female patients; (2) immunohistochemical staining (IHC) of ER was negative; (3) IHC of PR was negative; (4) IHC of HER2 and HER2 FISH test were negative; or IHC of HER2 was equivocal or not available but HER2 FISH test was negative; and (5) complete mRNA expression information. The total 139 samples with a next generation sequencing date were assessed for correlation with genes encoding VISTA(VSIR), CD3(CD3G), CD4(CD4), CD8(CD8A), and CD19(CD19). The date ID of the 139 TNBCs from the TCGA was shown in **Supplementary Table 2**.

#### Survival Analysis

Kaplan-Meier plotter website (http://kmplot.com/analysis) is a public database which evaluated the predictive effect of 54k genes on survival among 6,234 BC cases, followed up for an average of 69 months. It was used to analyze the relapse-free survival (RFS) and overall survival (OS) of basal-like BCs, based on the mRNA level of the VISTA coding gene C10orf54. Multiple genes incuding C10orf54 for VISTA and CD4 for CD4 were analyzed through the mean expression of proposed immune markers. The Affymetrix probe set IDs of VISTA and CD4 were 225372_at and 203547_at, respectively. The patients were analyzed for prognosis on the basis of gene expression.

#### Statistical Analysis

SPSS (version 19.0) was used for statistical analysis and GraphPad Prism (version 7.0) was used for plotting. Chi-square test was performed to assess the relevance of VISTA expression in the TME and clinicopathological parameters. The Spearman’s correlation test analyzed the association between the proportion of VISTA^+^ ICs, CD3^+^TILs, CD4^+^TILs, CD8^+^TILs, CD19^+^TILs, and total TILs. This was also done for VISTA^+^ TCs and TILs. Person’s correlation was employed to assess the mRNA expression of the above immune markers according to the TCGA database. The outcomes of this study were RFS and OS, which were plotted *via* the Kaplan-Meier method and compared using log-rank tests. The Cox regression models were performed to analyze prognostic factors and survival in TNBC. A two-sided p <0.05 was considered statistically significant. All the statistical methods corresponding to tables or figures were summarized in **Supplementary Table 3**.

## Results

### VISTA Expression and Clinicopathological Factors

A total of 254 patients were finally enrolled. The mean age was 49 ± 11 years (range 25–79 years). There were 145 (57.1%) patients younger than 50 years old, 120 (47.2%) patients had tumors ≤2 cm, and 120 (47.2%) patients had tumors between 2–5 cm. There were 108 (42.5%) patients with lymph node metastasis. Among them 53 (20.9%) patients had at least 4 involved nodes. According to the American Joint Committee on Cancer (AJCC), there were 116 (45.7%) and 55 (21.6%) patients with stage II and III, respectively.

**Figure 1:**
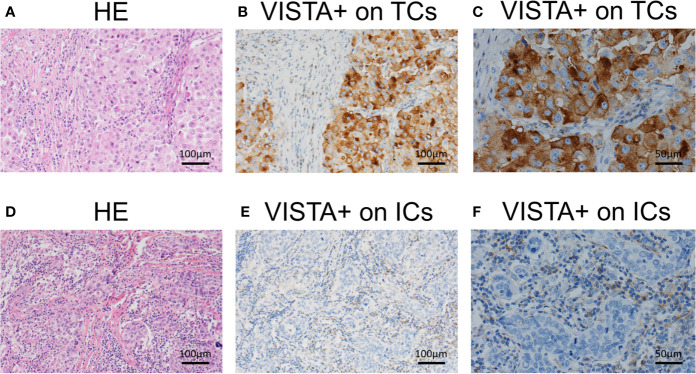
Representative immunohistochemical staining of V-domain Ig suppressor of T-cell activation (VISTA) on tumor cells and immune cells in triple-negative breast cancer. **(A)** Hematoxylin and eosin (HE) staining of the tumor microenvironment. **(B)** VISTA expression on tumor cells (Original magnifications×100). **(C)** VISTA expression on tumor cells (Original magnifications×200). **(D)** HE staining of tumor microenvironment. **(E)** VISTA expression on immune cells (Original magnifications×100). **(F)** VISTA expression on immune cells (Original magnifications×200).

VISTA expression was greater in ICs than in TCs. In both cell types, VISTA expression was exclusively cytoplasmic (**Figure 1**). VISTA was expressed by ICs in 223 cases (87.8%) with 143 “positive” cases (56.3%) showing high expression. Only 47 cases (18.5%) had VISTA expression in TCs in total. Only 24 cases were both positive for TCs and ICs while 88 cases tested negative for both. 119 cases were positive for ICs only, while 23 cases tested positive for TCs only.

VISTA-positive ICs were strongly correlated with no lymph node metastasis (p < 0.001), AJCC stage I and II (p = 0.001), and basal-like subtype (p < 0.001). VISTA-positive ICs did not show correlation with age, Ki-67, differentiation, and VISTA expression in TCs (**Table 1**). No correlation was established between VISTA-positive TCs and all the clinicopathological features (**Supplementary Table 4**).

**Table 1:** VISTA on immune cells and clinicopathological characteristics in TNBC.

Parameters			VISTA on immune cells				
			Negative		Positive		p value
	N	%	N	%	N	%	
**Age**							0.501
< 50 years	145	57.1	66	59.5	79	55.2	
≥ 50 years	109	42.9	45	40.5	64	44.8	
**Tumor stage**							0.907
pT1	120	47.2	52	46.8	68	47.6	
pT2	120	47.2	52	46.8	68	47.6	
pT3	9	3.6	4	3.6	5	3.5	
pT4	5	2	3	2.8	2	1.3	
**Lymph node**							<0.001
pN0	146	57.5	53	47.7	93	65	
pN1	55	21.7	22	19.8	33	23.1	
pN2	23	9.1	19	17.1	4	2.8	
pN3	30	11.7	17	15.4	13	9.1	
**AJCC stage**							0.001
I	83	32.7	30	27	53	37.1	
II	116	45.7	45	40.5	71	49.7	
III	55	21.6	36	32.5	19	13.2	
**Ki 67**							0.737
< 14%	39	15.4	18	16.2	21	14.7	
≥ 14%	215	84.6	93	83.8	122	85.3	
**Differentiation**							0.08
Well	2	0.8	0	0	2	1.4	
Moderate	67	26.4	36	32.4	31	21.7	
Poor	185	72.8	75	67.6	110	76.9	
**Basal like**							<0.001
Yes	223	87.8	86	77.5	137	95.8	
No	31	12.2	25	22.5	6	4.2	
**VISTA on TCs**							0.423
Positive	47	18.5	23	20.7	24	16.8	
Negative	207	81.5	88	79.3	119	83.2	

VISTA, V-domain Ig suppressor of T-cell activation; TNBC, triple-negative breast cancer; AJCC, American Joint Committee on Cancer; TC, tumor cell.

### VISTA Expression and Other Immune Markers in the TME

CD3^+^TILs, CD4^+^TILs, CD8^+^TILs, and CD19^+^TILs were also detected in the whole TNBC cohort. Representative IHC staining density of these immune marker were shown in **Supplementary Figure 1**. A high density of CD3^+^TILs, CD4^+^TILs, CD8^+^TILs, and CD19^+^TILs were found in 50.8% (129/254), 59.1% (150/254), 52.4% (133/254), and 54.7% (139/254) of TNBC tissues, respectively. VISTA expression in ICs showed a strong correlation with total TILs (p < 0.001), CD3^+^TILs (p = 0.009), CD4^+^TILs (p < 0.001), CD8^+^TILs (p = 0.021), and CD19^+^TILs (p < 0.001) (**Table 2**). No connection was established between VISTA expression in TCs and the four types of TILs (**Supplementary Table 5**).

**Table 2:** Correlation of VISTA on immune cells and other immune markers in TNBC.

Markers			VISTA on immune cells				
			Negative		Positive		p value
	N	%	N	%	N	%	
**TILs**							<0.001
< 5%	143	56.3	77	69.4	66	46.2	
≥ 5%	111	43.7	34	30.6	77	53.8	
**CD3**							0.009
< 20%	125	49.2	65	58.6	60	42	
≥ 20%	129	50.8	46	41.4	83	58	
**CD4**							<0.001
< 10%	104	40.9	67	60.4	37	25.9	
≥ 10%	150	59.1	44	39.6	106	74.1	
**CD8**							0.021
< 10%	121	47.6	62	55.9	59	41.3	
≥ 10%	133	52.4	49	44.1	84	58.7	
**CD19**							<0.001
< 1%	115	45.3	75	67.6	40	28	
≥ 1%	139	54.7	36	32.4	103	72	

VISTA, V-domain Ig suppressor of T-cell activation; TNBC, triple-negative breast cancer; TIL, tumor-infiltrating lymphocyte.

In the further Spearman’s correlation analysis, the proportion of VISTA-positive ICs were moderately and positively correlated with the percentage of CD4^+^TILs (ρ = 0.423, p < 0.001) and CD19^+^TILs (ρ = 0.465, p < 0.001), respectively. They were weakly related to CD3^+^TILs (ρ = 0.222, p < 0.001), CD8^+^TILs (ρ = 0.214, p < 0.001), and total TILs (ρ = 0.266, p < 0.001). Moreover, the percentage of CD4^+^TILs was strongly and positively related to CD3^+^TILs (ρ = 0.670, p < 0.001) and CD8^+^TILs (ρ = 0.632, p < 0.001). CD19^+^TILs were significantly related to CD3^+^TILs (ρ = 0.474, p < 0.001), CD4^+^TILs (ρ = 0.549, p < 0.001) and CD8^+^TILs (ρ = 0.487, p < 0.001). CD8^+^TILs and CD3^+^TILs were strongly correlated (ρ = 0.918, p < 0.001) (**Supplementary Table 6**).

**Figure 2:**
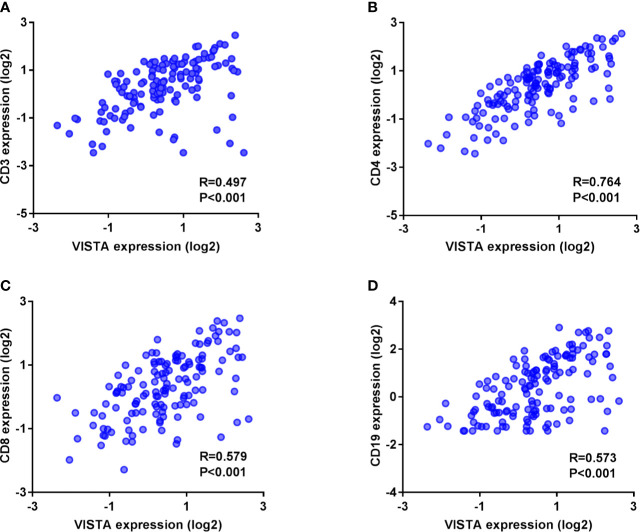
V-domain Ig suppressor of T cell activation (VISTA)-encoding co-expressed with genes that encode CD3 **(A)**, CD4 **(B)**, CD8 **(C)**, and CD19 **(D)** in triple-negative breast cancer samples from The Cancer Genome Atlas public database.

To further evaluate the relevance of VISTA expression and other immune markers in TNBC at the mRNA level, we assessed the correlation between the C10orf54 gene(encoding VISTA), CD3G(encoding CD3), CD4(encoding CD4), CD8A(encoding CD8), and CD19(encoding CD19) according to the mRNA expression of 139 TNBC patients downloaded from the TCGA database. The gene encoding VISTA was strongly and positively related to the gene encoding CD4 (R = 0.764, p < 0.001) (**Figure 2B**). It was also moderately related to the genes encoding CD8 (R = 0.579, p < 0.001) (**Figure 2C**), CD19 (R = 0.573, p < 0.001) (**Figure 2D**) and CD3 (R = 0.497, p < 0.001) (**Figure 2A**) (**Supplementary Table 7**).

### VISTA Expression on ICs Reveals Long-Term Survival in TNBC Patients

**Figure 3:**
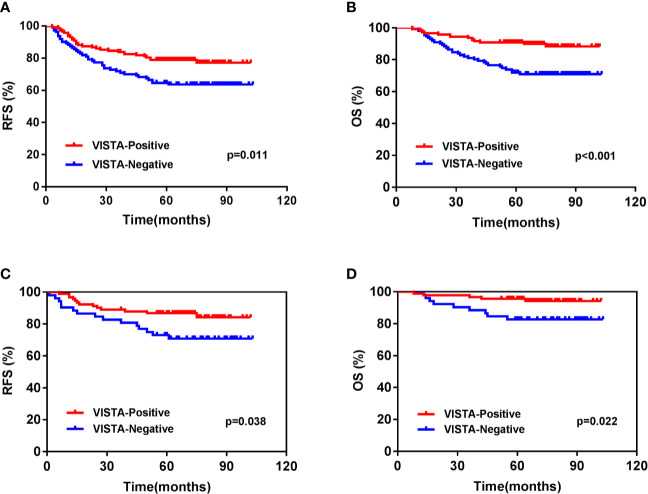
Kaplan-Meier survival analysis of V-domain Ig suppressor of T cell activation (VISTA) expression on immune cells in triple-negative breast cancer patients. **(A)** Relapse-free survival (RFS) of the whole cohort. **(B)** Overall survival (OS) of the whole cohort. **(C)** RFS of T1-2N0 patients. **(D)** OS of T1-2N0 patients.

For the entire cohort, the 5-year RFS and OS rates were 72.7 and 82.6%, respectively. The VISTA-positive ICs group had a more favorable 5-years RFS (79.0 vs. 64.6%, p = 0.011) (**Figure 3A**) and OS (90.0 vs. 72%, p < 0.001) (**Figure 3B**) than the negative group (**Supplementary Table 8**). The correlation between mRNA level and survival was further analyzed. The Kaplan-Meier plots showed that basal-like BCs with high level of C10orf54 had a better RFS (HR = 0.64, p = 0.007) (**Supplementary Figure 2A**), but OS remained the same (HR = 0.95, p = 0.860) (**Supplementary Figure 2B**). Multivariate Cox regression analysis of the TNBC cohort showed that VISTA-positive ICs (p = 0.012) and AJCC stage (p = 0.001) independently predicted the OS, while age, Ki-67, and tumor differentiation did not (**Table 3**).

**Table 3:** Multivariate analysis for patients with TNBC.

	Relapse-free survival		Overall survival		
	HR (95% CI)	p value	HR (95% CI)	p value	
**VISTA on ICs**		0.158		0.012	
Negative	1		1		
Positive	0.698 (0.423–1.151)		0.436 (0.228–0.831)		
**Age**		0.999		0.709	
< 50 years	1		1		
≥ 50 years	1.000 (0.619–1.615)		1.119 (0.620–2.019)		
**AJCC stage**		<0.001		0.001	
I	1		1		
II	1.884 (0.959–3.703)	0.066	2.181 (0.863–5.508)	0.099	
III	4.182 (2.066–8.466)	<0.001	5.217 (2.068–13.164)	<0.001	
**Ki 67**		0.2		0.366	
< 14%	1		1		
≥ 14%	1.683 (0.760–3.727)		1.549 (0.600–3.996)		
**Differentiation**		0.922		0.951	
Moderate+Well	1		1		
Poor	1.028 (0.594–1.777)		1.021 (0.522–1.996)		

TNBC, triple-negative breast cancer; VISTA, V-domain Ig suppressor of T-cell activation; IC, immune cell; AJCC, American Joint Committee on Cancer; CI, confidence interval.

On further subgroup analysis, the predictive effect of VISTA expression in ICs was significant for OS in T1 (p = 0.032), T2 (p = 0.005), and N0 (p = 0.020) patients. This effect was also significant for RFS in T2 (p = 0.017) and N0 (p = 0.030) patients (**Supplementary Table 8**). We further analyzed the T1-2N0 subgroups. The results showed that the VISTA-positive ICs group had a longer 5-years RFS (86.8 vs. 73.1%, p = 0.038) (**Figure 3C**) and OS (95.6 vs. 82.7%, p = 0.022) (**Figure 3D**) than the negative groups. Multivariate analysis showed that VISTA-positive ICs was the only prognostic indicators for both RFS (p = 0.044) and OS (p = 0.024) (**Table 4**) in T1-2N0 TNBC patients.

**Table 4:** Multivariate analysis for patients with T1-2N0 TNBC.

	Relapse-free survival				Overall survival			
	HR (95% CI)		p value		HR (95% CI)		p value	
**VISTA on ICs**			0.044				0.024	
Negative	1				1			
Positive	0.462 (0.218–0.979)				0.281 (0.093–0.848)			
**Age**			0.469				0.303	
< 50 years	1				1			
≥ 50 years	1.328 (0.617–2.858)				1.758 (0.601–5.147)			
**Tumor stage**			0.115				0.302	
T1	1				1			
T2	1.851 (0.861–3.982)				1.764 (0.601–5.179)			
**Ki 67**			0.409				0.966	
< 14%	1				1			
≥ 14%	1.677 (0.492–5.717)				1.034 (0.220–4.854)			
**Differentiation**			0.924				0.612	
Moderate+Well	1				1			
Poor	1.043 (0.436–5.717)				1.399 (0.382–5.115)			

TNBC, triple-negative breast cancer; VISTA, V-domain Ig suppressor of T-cell activation; IC, immune cell; AJCC, American Joint Committee on Cancer; CI, confidence interval.

### Classification of the TME According to VISTA^+^ ICs and CD4^+^TILs

**Figure 4:**
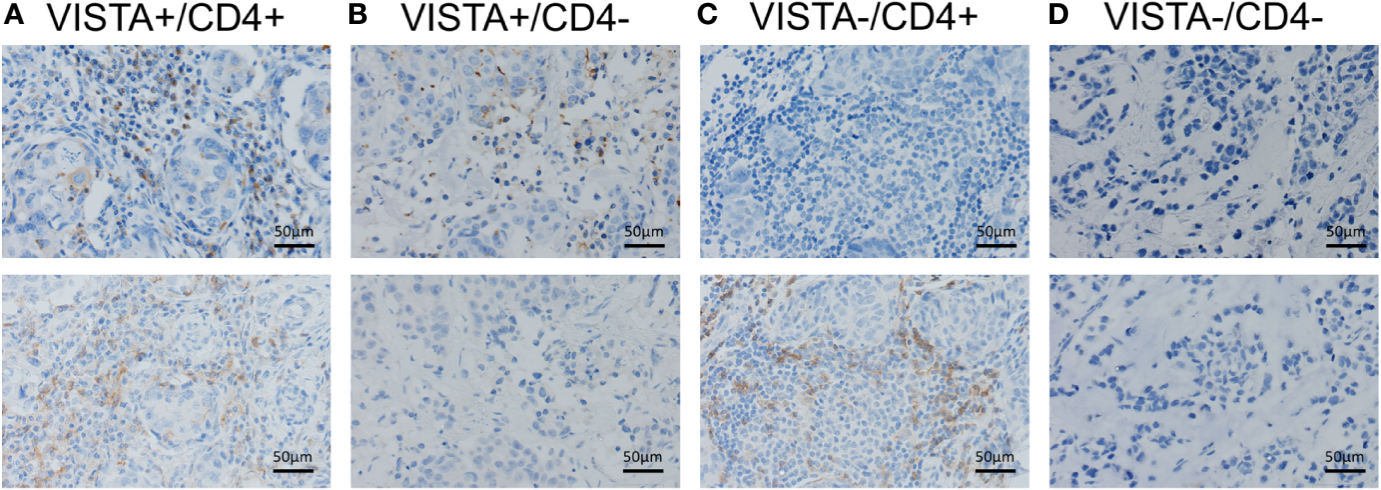
Classification of the triple-negative breast cancer immune microenvironment according to the expression of V-domain Ig suppressor of T cell activation (VISTA) on immune cells and CD4^+^ tumor-infiltrating lymphocytes (TILs). **(A)** VISTA+/CD4+. **(B)** VISTA+/CD4-. **(C)** VISTA-/CD4+. **(D)** VISTA-/CD4-.

**Figure 5:**
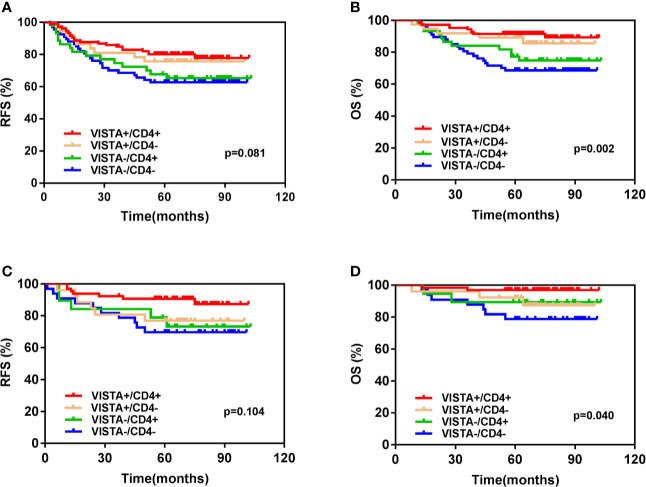
Kaplan-Meier survival analysis based on different immune subtypes according to V-domain Ig suppressor of T cell activation (VISTA) and CD4 of **(A)** Relapse-free survival (RFS) of the whole cohort. **(B)** Overall survival (OS) of the whole cohort. **(C)** RFS of T1-2N0 patients. **(D)** OS of T1-2N0 patients.

We classified the TNBC immune microenvironment into four subtypes based on the expression of VISTA in ICs and the density of CD4^+^ TILs: VISTA+/CD4+ (41.7%, 106/254), VISTA+/CD4- (14.6%, 37/254), VISTA-/CD4+ (17.3%, 44/254), and VISTA-/CD4- (26.4%, 67/254) (**Figure 4**). Kaplan–Meier survival analysis indicated that patients with both VISTA-positive ICs and high CD4^+^TILs density had a significantly increased 5-year OS rate than others (VISTA+/CD4+ vs. VISTA +/CD4- vs. VISTA-/CD4+ vs. VISTA-/CD4-: 91.5 vs. 89.2 vs. 77.3 vs. 68.7%, p = 0.002) (**Figure 5B**).A similar trend was observed in 5-years RFS without statistical significance (VISTA+/CD4+ vs. VISTA +/CD4- vs. VISTA-/CD4+ vs. VISTA-/CD4-: 80.1 vs. 75.7 vs. 67.7 vs. 62.7%, p = 0.081) (**Figure 5A**). In the T1-2N0 subgroups, similar trends and statistical differences were observed in RFS (p = 0.104) (**Figure 5C**) and OS (p = 0.040) (**Figure 5D**).

We assessed the prognostic effect of the co-expression of mRNA encoding for VISTA and CD4 in basal-like BC patients *via* a multigene classifier and survival analysis. Patients with high levels of the co-expression of C10orf54 and CD4 had significantly prolonged RFS(HR = 0.36, p < 0.001) (**Supplementary Figure 2C**) and OS (HR = 0.49, p = 0.034) (**Supplementary Figure 2D**).

## Discussion

VISTA is a new immune checkpoint that has been studied previously in cell lines, murine models (17), and human cancer cohorts, including breast cancer (29) and other malignances (19, 28, 33–39). However, there are still few reports about VISTA, with no large-scale studies in a TNBC cohort. In this research, we explored the distribution of VISTA in the TME, its correlation with other immune markers, and its prognostic value in a large-scale TNBC cohort. We found that VISTA was expressed in both ICs and TCs. High VISTA expression in ICs was significantly related to survival benefits in the whole TNBC cohort, especially for T1-2N0 patients. In the TME, VISTA expression in ICs was positively related to T lymphocyte markers (CD3, CD4, and CD8) and B lymphocyte marker (CD19). Patients with VISTA-positive ICs and high CD4^+^TILs density had significantly longer survival than those in other subgroups.

VISTA expression was greater in ICs than in TCs, with membrane and/or cytoplasmic staining. Previous experiments suggested that VISTA functioned as both a ligand on antigen-presenting cells (APCs) and a receptor on T cells (17). In vitro studies on cell lines and mouse models demonstrated that VISTA was principally expressed in leukocytes infiltrating the TME (18). Zong et al. (29) suggested that in BCs, the expression of VISTA in ICs was higher than that in TCs, but the overall positive rate was lower than that of this study. Studies on gastric cancer (34) and non–small cell lung cancer (NSCLC) (19) suggested that VISTA was expressed more in ICs than in TCs. Loeser et al (28). showed that VISTA was rarely present in esophageal adenocarcinoma (1.2%). However, in hepatocellular carcinoma (35) and ovarian cancer (39), the expression rates of VISTA in TCs and ICs were similar. The variable level of expression may be caused by the differences of immunogenicity among different human malignancies.

In the cross-table analysis, VISTA-positive ICs were associated with nodal negative patients (p < 0.001) and early AJCC stage (I/II) (p = 0.001). However, it was not correlated with age, Ki-67 and differentiation. This indicated that VISTA expressed in ICs affected local tumor progression in the TME. VISTA expression may also have been caused by the decrease in tumor immunogenicity to achieve immune evision and disease development according to the theory of cancer immunoediting. This mechanism leads to a decrease in tumor infiltrating ICs and VISTA expression.

Previous studies reported VISTA as a negative immune checkpoint, which potentially restrained T-cells proliferation and activation. However, the relevance of VISTA expression in the TME in survival remains controversial. In this study, TNBC patients with VISTA-positive ICs, especially the T1-2N0 stage patients and basal-like subgroup patients had a significantly favorable prognosis in terms of RFS and OS Recent studies on esophageal adenocarcinoma (28) and breast cancer (29) also confirmed that high levels of VISTA in ICs was related to a better prognosis. Research on NSCLC (19) showed that a high VISTA expression in the tumor area was related to prolonged survival, while in most cases, it was only expressed in the tumor stroma. Meanwhile, studies on hepatocellular carcinoma (35) and high-grade serous ovarian cancer (39) showed that VISTA expression in TCs was significantly related to a favorable survival. However, studies on gastric cancer (34) and oral squamous cell carcinoma (37) showed no relationship between VISTA expression and survival. A study on cutaneous melanoma (38) discovered a negative correlation between VISTA expression in ICs and prognosis. All these findings showed that the mechanism of VISTA expression in TCs and ICs behaved differently, and its relationship with prognosis varied across the different cancers.

A positive connection was also found between VISTA expression and TILs, especially the CD4^+^TILs. Similar results were obtained at mRNA level. Previous studies demonstrated that VISTA was constitutively expressed in naïve CD4^+^T cell as a co-inhibitory T cell receptor, limiting CD4^+^T cell activation and function (22, 40). A recent study also showed that VISTA played a critical role in regulating naïve T cell quiescence and peripheral tolerance. However, this was lost under inflammatory conditions (23). This may explain why the high expression of VISTA in ICs was closely related to CD4 ^+^TILs in TNBC. This was consistent with a study on esophageal adenocarcinoma (28) which showed a strong co-expression between VISTA and CD4. However, it contradicts the study on hepatocellular carcinoma (35) which showed a strong association between VISTA and CD8^+^TILs rather than CD4^+^TILs.

Previous studies demonstrated that VISTA in both T cells and APCs contributed to the suppression of immunity.(17.22) However, in this study, we found that TNBC patients with both VISTA-positive ICs and high density of CD4^+^TILs had a significantly better prognosis than patients with only one or no increased index. The tumor associated inflammatory environment may have relieved naïve T cells suppression by VISTA. A ligand may have also bound to VISTA on the CD4^+^T cell and stimulated cell differentiation into type 1 T helper cells, which play an important role in anti-tumor immunity.

Recent studies showed that VISTA expression increased after the blockade of PD-1 in metastatic melanoma (26) and of CTLA-4 in prostate cancer (25),respectively. This indicated that VISTA may play a significant role in immunotherapy tolerance. Further studies exploring the potential relationship between VISTA and the PD-1 axis or other inhibitors are under way. There are also some clinical trials about VISTA. NCT02671955 is a phase 1 trial of JNJ-61610588 (a VISTA inhibitor) in human progressed cancers. NCT02812875 is another phase 1 clinical trial of CA-170 (inhibit PD-L1/PD-L2/VISTA) in human advanced solid tumors. We look forward to the results of these experiments, which will help understand the immune mechanism of VISTA in the TME and its synergistic anti-tumor effect with other immune checkpoints.

This research had limitations. First, it was a retrospective study with a limited sample size. Second, owing to intra-tumoral heterogeneity, TMA may not have been able to completely reflect the TME. Third, immunofluorescence may accurately show the co-expression of VISTA and lymphocyte markers (CD3, CD4, CD8, and CD19).

## Conclusion

Our study revealed that VISTA expression was greater in ICs than in TCs in the TNBC cohort. It was also associated with prolonged RFS and OS, especially among T1-2N0 stage patients. Expression of VISTA in ICs was closely correlated with tumor-infiltrated ICs. We look forward to more studies on the immunoregulation mechanism of VISTA as this will provide more options for TNBC immunotherapy.

## Data Availability Statement

The datasets presented in this study can be found in online repositories. The names of the repository/repositories and accession number(s) can be found below: Breast Invasive Carcinoma (TCGA, PanCancer Atlas) (https://www.cbioportal.org/study/summary?id=brca_tcga_pan_can_atlas_2018).

## Ethics Statement

The studies involving human participants were reviewed and approved by Human Research of Peking Union Medical College Hospital. The patients/participants provided their written informed consent to participate in this study. Written informed consent was obtained from the individual(s) for the publication of any potentially identifiable images or data included in this article.

## Author Contributions

XC and QS conceived, designed, and supervised the whole study. XC performed experiments, collected and analyzed data; and wrote, edited, and reviewed the manuscript. XR and HW performed the experiments and assessed the immunostaining results. YZ, FM, and YL evaluated and analyzed database from the public website and the whole TNBC cohort. They also guided the statistical methods. All authors contributed to the article and approved the submitted version.

## Funding

This study is supported by the Fundamental Research Funds for the Central Universities (3332020001), and the funders are not involved in this study and have no interest connection.

## Conflict of Interest

The authors declare that the research was conducted in the absence of any commercial or financial relationships that could be construed as a potential conflict of interest.
