# Supplementary material for: VISTA Expression on Immune Cells Correlates With Favorable Prognosis in Patients With Triple-Negative Breast Cancer
Source: Front Oncol. 2021 Jan 11;10:583966. doi: 10.3389/fonc.2020.583966 (PMC7829913; doi:10.3389/fonc.2020.583966)

Supplementary Table 1.Details of primary antibodies and immunohistochemistry dilutions.

| **Antigen** | **Clone** | **Catalogue** | **Dilution** | **Vendor** |
|---|---|---|---|---|
| VISTA | D1L2G | 64953 | 1:200 | Cell Signalling Technology, United States of America |
| CD3 | LN10 | PA0553 | Prediluted | Leica Microsystems, Shanghai of China |
| CD4 | 4B12 | PA0427 | Prediluted | Leica Microsystems, Shanghai of China |
| CD8 | 4B11 | PA0183 | Prediluted | Leica Microsystems, Shanghai of China |
| CD19 | EP169 | ZA-0569 | Prediluted | Zhongshan Golden Bridge Biotechnology CO.LTD, Beijing of China |

SupplementaryTable 2. The 139 TNBC patients ID from The Cancer Genome Atlas Provisional dataset.

| PATIENT_ID | PATIENT_ID | PATIENT_ID | PATIENT_ID | PATIENT_ID |
|---|---|---|---|---|
| TCGA-AC-A7 | TCGA-E9-A5 | TCGA-C8-A2 | TCGA-HN-A2 | TCGA-A2-A0 |
| TCGA-E2-A57 | TCGA-OL-A9 | TCGA-AN-A | TCGA-D8-A1 | TCGA-GM-A |
| TCGA-A7-A4 | TCGA-E2-A1 | TCGA-E2-A1 | TCGA-A7-A2 | TCGA-AN-A |
| TCGA-GI-A2C | TCGA-BH-A0 | TCGA-A7-A6 | TCGA-OL-A6 | TCGA-AO-A |
| TCGA-E2-A1L | TCGA-D8-A2 | TCGA-EW-A | TCGA-E2-A1 | TCGA-GM-A |
| TCGA-BH-A1 | TCGA-BH-A0 | TCGA-OL-A6 | TCGA-BH-A0 | TCGA-E2-A1 |
| TCGA-AQ-A5 | TCGA-B6-A4 | TCGA-C8-A3 | TCGA-GM-A | TCGA-E2-A1 |
| TCGA-A7-A5 | TCGA-AN-A0 | TCGA-AO-A | TCGA-A7-A4 | TCGA-C8-A1 |
| TCGA-AO-A0 | TCGA-D8-A2 | TCGA-A2-A0 | TCGA-A8-A0 | TCGA-BH-A |
| TCGA-A8-A07 | TCGA-E2-A1 | TCGA-AR-A1 | TCGA-D8-A1 | TCGA-AR-A |
| TCGA-AC-A2 | TCGA-C8-A1 | TCGA-A1-A0 | TCGA-AR-A1 | TCGA-S3-AA |
| TCGA-A7-A0 | TCGA-A2-A3 | TCGA-EW-A | TCGA-BH-A0 | TCGA-AR-A |
| TCGA-AO-A1 | TCGA-AR-A0 | TCGA-E2-A1 | TCGA-BH-A0 | TCGA-OL-A |
| TCGA-BH-A0 | TCGA-A2-A0 | TCGA-D8-A1 | TCGA-BH-A0 | TCGA-D8-A2 |
| TCGA-E2-A15 | TCGA-A2-A0 | TCGA-A2-A0 | TCGA-A7-A6 | TCGA-EW-A |
| TCGA-AR-A1 | TCGA-A2-A1 | TCGA-A7-A6 | TCGA-LL-A7 | TCGA-AQ-A |
| TCGA-B6-A40 | TCGA-OL-A5 | TCGA-GM-A | TCGA-A2-A3 | TCGA-OL-A |
| TCGA-E2-A1L | TCGA-EW-A | TCGA-A8-A0 | TCGA-AO-A1 | TCGA-A2-A0 |
| TCGA-AR-A2 | TCGA-AO-A0 | TCGA-BH-A0 | TCGA-A2-A3 | TCGA-EW-A |
| TCGA-A2-A3 | TCGA-E2-A1 | TCGA-E2-A1 | TCGA-GM-A | TCGA-AR-A |
| TCGA-LL-A74 | TCGA-AR-A0 | TCGA-AN-A | TCGA-E2-A5 | TCGA-EW-A |
| TCGA-A2-A0 | TCGA-D8-A1 | TCGA-EW-A | TCGA-AR-A2 | TCGA-A2-A0 |
| TCGA-EW-A1 | TCGA-B6-A4 | TCGA-C8-A2 | TCGA-BH-A0 | TCGA-E2-A1 |
| TCGA-AO-A1 | TCGA-C8-A1 | TCGA-A2-A0 | TCGA-GM-A | TCGA-LL-A5 |
| TCGA-AN-A0 | TCGA-A1-A0 | TCGA-E2-A1 | TCGA-AO-A1 | TCGA-S3-AA |
| TCGA-A2-A0 | TCGA-D8-A1 | TCGA-D8-A1 | TCGA-A2-A0 | TCGA-EW-A |
| TCGA-A7-A0 | TCGA-EW-A | TCGA-E2-A1 | TCGA-C8-A2 | TCGA-LL-A4 |
| TCGA-AN-A0 | TCGA-AC-A2 | TCGA-AC-A6 | TCGA-BH-A0 | |

TNBC, triple-negative breast cancer.

SupplementaryTable 3.Details of statistical methods used in tables and figures.

| Statistical methods | Corresponding tables or figures |
|---|---|
| Chi-square test | Table 1. Table 2. Supplementary Table 4. Supplementary Table 5. |
| Spearman's correlation test | Supplementary Table 6. |
| Person's correlation test | Figure2. Supplementary Table 7. |
| Kaplan-Meier method with log-rank tests | Figure 3. Figure 5. Supplementary Table 8. |
| Cox regression models analysis | Table 3. Table 4. |

SupplementaryTable 4.VISTA on tumor cells and clinicopathological characteristics in TNBC.

| **Parameters** | | | **VISTA on tumor cells** | | | | |
|---|---|---|---|---|---|---|---|
| | | | Negative | | Positive | | p value |
| | N | % | N | % | N | % | |
| **Age** | | | | | | | 0.786 |
| <50 years | 145 | 57.1 | 119 | 57.5 | 26 | 55.3 | |
| ≥50 years | 109 | 42.9 | 88 | 42.5 | 21 | 44.7 | |
| **Tumor stage** | | | | | | | 0.272 |
| pT1 | 120 | 47.2 | 103 | 49.8 | 17 | 36.2 | |
| pT2 | 120 | 47.2 | 94 | 45.4 | 26 | 55.2 | |
| pT3 | 9 | 3.5 | 7 | 3.4 | 2 | 4.3 | |
| pT4 | 5 | 2.1 | 3 | 1.4 | 2 | 4.3 | |
| **Lymph node** | | | | | | | 0.792 |
| pN0 | 146 | 57.5 | 117 | 56.5 | 29 | 61.7 | |
| pN1 | 55 | 21.7 | 46 | 22.2 | 9 | 19.1 | |
| pN2 | 23 | 9.1 | 18 | 8.7 | 5 | 10.6 | |
| pN3 | 30 | 11.7 | 26 | 12.6 | 4 | 8.6 | |
| **AJCC stage** | | | | | | | 0.326 |
| I | 83 | 32.7 | 71 | 34.3 | 12 | 25.5 | |
| II | 116 | 45.7 | 90 | 43.5 | 26 | 55.3 | |
| III | 55 | 21.6 | 46 | 22.2 | 9 | 19.2 | |
| **Ki 67** | | | | | | | 0.090 |
| <14% | 39 | 15.4 | 28 | 13.5 | 11 | 23.4 | |
| ≥14% | 215 | 84.6 | 179 | 86.5 | 36 | 76.6 | |
| **Differentiatio** | | | | | | | 0.307 |
| Well | 2 | 0.8 | 1 | 0.5 | 1 | 2.1 | |
| Moderate | 67 | 26.4 | 52 | 25.1 | 15 | 31.9 | |
| Poor | 185 | 72.8 | 154 | 74.4 | 31 | 66.0 | |
| **Basal like** | | | | | | | 0.533 |
| Yes | 223 | 87.8 | 183 | 88.4 | 40 | 85.1 | |
| No | 31 | 12.2 | 24 | 11.6 | 7 | 14.9 | |
| **VISTA on IC** | | | | | | | 0.423 |
| Positive | 143 | 56.3 | 119 | 57.5 | 24 | 51.1 | |
| Negative | 111 | 43.7 | 88 | 42.5 | 23 | 48.9 | |

VISTA, V-domain Ig suppressor of T-cell activation; TNBC, triple-negative breast cancer; AJCC, American Joint Committee on Cancer; IC, immune cell

SupplementaryTable 5.Correlation of VISTA on tumor cells and other immune markers in TNBC.

| **Markers** | | | **VISTA on tumor cells** | | | | |
|---|---|---|---|---|---|---|---|
| | | | Negative | | Positive | | p value |
| | N | % | N | % | N | % | |
| **TILs** | | | | | | | 0.861 |
| <5% | 143 | 56.3 | 116 | 56.0 | 27 | 57.4 | |
| ≥5% | 111 | 43.7 | 91 | 44.0 | 20 | 42.6 | |
| **CD3** | | | | | | | 0.491 |
| <20% | 125 | 49.2 | 104 | 50.2 | 21 | 44.7 | |
| ≥20% | 129 | 50.8 | 103 | 49.8 | 26 | 55.3 | |
| **CD8** | | | | | | | 0.653 |
| <10% | 121 | 47.6 | 100 | 48.3 | 21 | 44.7 | |
| ≥10% | 133 | 52.4 | 107 | 51.7 | 26 | 55.3 | |
| **CD4** | | | | | | | 0.683 |
| <10% | 104 | 40.9 | 86 | 41.5 | 18 | 38.3 | |
| ≥10% | 150 | 59.1 | 121 | 58.5 | 29 | 61.7 | |
| **CD19** | | | | | | | 0.165 |
| <1% | 115 | 45.3 | 98 | 47.3 | 17 | 36.2 | |
| ≥1% | 139 | 54.7 | 109 | 52.7 | 30 | 63.8 | |

VISTA, V-domain Ig suppressor of T-cell activation; TNBC, triple-negative breast cancer;

TIL, tumor-infiltrating lymphocyte.

SupplementaryTable 6. Correlation between percentage of VISTA-posivite ICs and TILs by Spearman's correlation analyses in TNBC.

| Variables (%) | VISTA on ICs | VISTA on TCs | CD3+TILs | CD4+TILs | CD8+TILs | CD19+TILs | TILs |
|---|---|---|---|---|---|---|---|
| VISTA on ICs | 1 | | | | | | |
| VISTA on TCs | 0.013 | 1 | | | | | |
| CD3+TILs | 0.222* | 0.101 | 1 | | | | |
| CD4+TILs | 0.423* | 0.067 | 0.670* | 1 | | | |
| CD8+TILs | 0.214* | 0.075 | 0.918* | 0.632* | 1 | | |
| CD19+TILs | 0.465* | 0.049 | 0.474* | 0.549* | 0.487* | 1 | |
| TILs | 0.266* | 0.014 | 0.667* | 0.531* | 0.649* | 0.601* | 1 |

*P<0.001

VISTA, V-domain Ig suppressor of T-cell activation; IC, immune cell; TC, tumor cell; TIL, tumor-infiltrating lymphocyte; TNBC, triple-negative breast cancer.

SupplementaryTable 7. Correlation between VISTA mRNA and CD3, CD4, CD8 and CD19 mRNA by Person's correlation analyses in 139 TNBC from the The Cancer Genome Atlas Provisional dataset.

| Variables | VISTA | CD3 | CD4 | CD8 | CD19 |
|---|---|---|---|---|---|
| VISTA | 1 | | | | |
| CD3 | 0.497* | 1 | | | |
| CD4 | 0.764* | 0.709* | 1 | | |
| CD8 | 0.579* | 0.839* | 0.689* | 1 | |
| CD19 | 0.573* | 0.732* | 0.621* | 0.711* | 1 |

*P<0.001

VISTA, V-domain Ig suppressor of T-cell activation; TNBC, triple-negative breast cancer.

SupplementaryTable 8.Relationships between VISTA expression on ICs and 5-year RFS/OS in TNBC.

| Characteristics | | **5-year Relapse-free survival (%)** | | | **5-year Overall survival (%)** | | |
|---|---|---|---|---|---|---|---|
| | | VISTA on ICs Negative | VISTA on ICs Positive | p | VISTA on ICs Negative | VISTA on ICs Positive | p |
| Whole group | | 64.6 | 79.0 | **0.011** | 72.0 | 90.0 | **<0.001** |
| Basal like | | 63.6 | 78.8 | **0.020** | 70.9 | 90.5 | **<0.001** |
| T1-2N0 | | 73.1 | 86.8 | **0.038** | 82.7 | 95.6 | **0.022** |
| Tumor stage | T1 | 75.0 | 83.8 | 0.189 | 78.6 | 94.1 | **0.032** |
| | T2 | 57.1 | 77.9 | **0.017** | 69.2 | 89.7 | **0.005** |
| | T3 | - | - | - | - | - | - |
| | T4 | - | - | - | - | - | - |
| Lymph node | N0 | 71.7 | 86 | **0.030** | 81.1 | 94.6 | **0.020** |
| | N1 | 68.2 | 78.8 | 0.381 | 72.4 | 93.9 | 0.103 |
| | N2 | 61.5 | - | - | 73.7 | - | - |
| | N4 | 41.2 | 38.5 | 0.901 | 41.2 | 69.2 | 0.120 |
| AJCC stage | I | 79.4 | 88.6 | 0.295 | 86.7 | 98.1 | 0.107 |
| | II | 62.2 | 81.7 | **0.031** | 73.3 | 91.5 | **0.020** |
| | III | 51.7 | 42.1 | 0.473 | 58.3 | 68.4 | 0.443 |

VISTA, V-domain Ig suppressor of T-cell activation; AJCC, American Joint Committee on Cancer; IC, immune cell; RFS, relapse-free survival; DSS, disease-specific survival; TNBC, triple-negative breast cancer.

**Supplementary Figure 1**.Representative immunohistochemical staining of (a) CD3, (b)CD4, (c)CD8 and (d) CD19 in tumor-infiltrating lymphocytes (TILs) in triple-negative breast cancer.

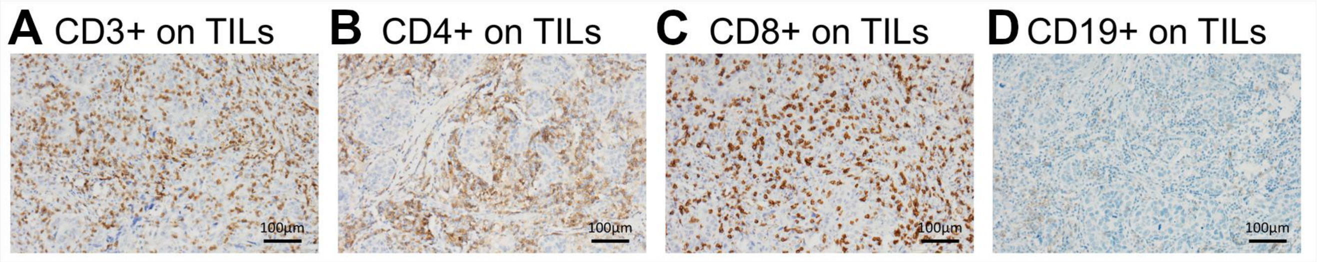


**Supplementary Figure 2**.High expression of VISTA mRNA (C10orf54) correlated with favorable (a)relapse-free survival (RFS) and (b)overall survival(OS) in basal-like breast cancer. High expression of VISTA mRNA (C10orf54) and CD4(CD4) using multigene classifier showed prolonged (c) RFS and (d) OS.

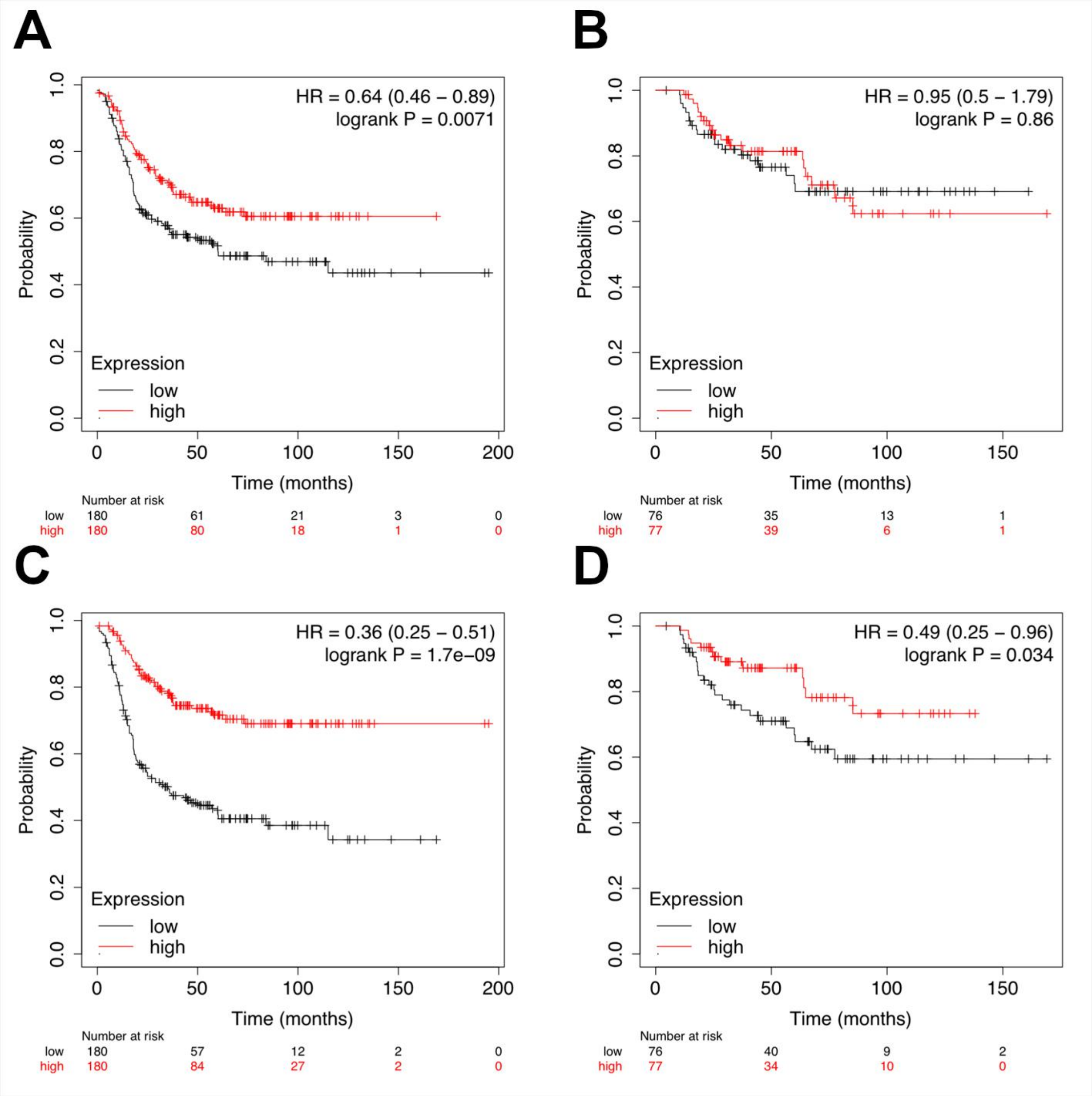

Supplement: Supplementary file 1 [file DataSheet_1.pdf]
